# Previous flight facilitates partner finding in female crickets

**DOI:** 10.1038/s41598-020-78969-w

**Published:** 2020-12-18

**Authors:** Maxim Mezheritskiy, Dmitry Vorontsov, Dmitry Lapshin, Varvara Dyakonova

**Affiliations:** 1 Koltzov Institute of Developmental Biology, Russian Academy of Sciences, Vavilov Str. 26, 119334 Moscow, Russia; 2 Institute for Information Transmission Problems, Russian Academy of Sciences, Moscow, Russia

**Keywords:** Zoology, Animal behaviour

## Abstract

In the cricket *Gryllus bimaculatus,* flying occurs soon after the last imaginal molt and precedes the mating behavior in natural conditions. Here, we tested the hypothesis that flying may improve subsequent behavioral performance in a novel environment in female crickets. We developed a behavioral set-up to test female cricket responsiveness to male calling song as well as their ability to locate and find the source of the song. The male song was produced by a loudspeaker hidden behind the fabric wall of a spacious square arena. Forced flight prior to the test promoted female sexual searching behavior in the novel environment. After the flight, more females reached the hidden source zone, spent more time near the source and finally more of them climbed over the wall section immediately in front of the hidden loudspeaker. At the same time, their behavior in the arena did not differ from the control group when the calling song was not delivered, suggesting that flight exerts its behavioral effects by influencing sexual motivation. Our results support the suggestion that preceding intense locomotion facilitates sexual searching behavior of females in a novel environment.

## Introduction

In many insects, flight behavior and associated change of territory precede mating behavior^1^. Novel and unpredictable conditions decrease the chances of survival and demand higher cognitive and physical abilities in order to navigate, avoid possible risks or reach a goal. Little is known on whether flying affects sexual and cognitive behavior in insects. One might expect augmentation of these functions since increased sexual activity could benefit intruders by compensating for their otherwise lower competitiveness in a novel territory^1–4^. On the other hand, intense locomotion generates high energetic costs^5^, while courtship and mating behaviors require additional costly metabolic investments. Moreover, active behaviors attract predators and thus may be particularly risky in an unfamiliar environment^6^. It still remains largely unknown whether possible benefits outweigh the costs of augmented mating activity and to what extent the solution to this compromise depends on the ecology of the species.

The hypothetical oogenesis-flight syndrome, posits reciprocal regulation of these energy-demanding behaviors in females, has been discussed and evaluated in various insect species^1,7–13^. The evidence for this is, however, contradictory, being confirmed for some animal species^7,8,13^, but not others^9–12^. Although the data are still sparse, activating effects of flying on agonistic^2,3^ and courtship^4^ behaviors were obtained in crickets.

Flying behavior is an example of within and between habitat dispersal in field crickets^14^. In *Gryllus bimaculatus,* flying usually occurs within the first week after the last imaginal molt. Later, some flight muscles degrade^8,15^, however, older crickets preserve the ability to activate the central pattern generator for flying in the air stream when raised in captivity. This advantage has already been successfully used to demonstrate flight-induced behavioral modulation and elucidate the underlying neurochemical mechanisms^2–4,16,17^. Thus, flying has been reported to restore the ability of males to fight^2^, to intensify aggression^3^ and courtship singing^4^, as well as to increase their mating success^4^. Neuromodulators octopamine and serotonin are suggested to play a role in the above effects^3,16–19^. Although an inverse correlation between the flight ability and oogenesis has been found in this animal^8^, the influence of flight on sexual behavior and orientation in novel environments in females remains unknown.

Here, we developed a new behavioral approach to test female cricket responsiveness to male calling song, as well as the ability to locate and find the hidden source of the song in the spacious arena, that had been unfamiliar to the crickets previously. One important detail of the method was that the animals could freely choose when to leave their home containers in the novel environment, that, as we believe, reduced the number of stressing factors that could affect the behavior in the arena.

We found that flight prior to the test promoted female sexual searching behavior. After the flight, more females ran towards the hidden source of a calling song, kept closer to the area near that source, visited the area more frequently and spent more time in it. After flying, more females attempted to reach the hidden sound source by climbing over the retaining wall. At the same time, their behavior in the arena did not differ from the control group when the calling song was not delivered, suggesting that flight exerts its behavioral effects by influencing sexual motivation. We consider the obtained effects as manifestations of proactive physiological adjustment to a novel environment that is likely to be reached as a result of flying.

## Results

We first tested whether the designed experimental paradigm can be used to investigate female cricket sexual and phonotaxis behavior in the novel environment. In this series, we used females that were isolated and unhandled for at least 24 h before the experiment (n = 13). All of them left their home containers in the novel spacious arena with a source of male calling song hidden behind the fabric wall of the arena. 6 of 13 females left the experimental arena before the end of the experiment and did not reach the 'Male zone' near the source. However, 7 of 13 crickets reached the “Male zone” and returned to it several times, thus demonstrating clear attraction to the area near the speaker. One of them climbed up the wall in the 'Male zone' behind which the speaker was hidden. Therefore, we concluded that the designed setup can be used to investigate the possible influence of flying on sexual motivation and orientation in the novel territory.

**Figure 1:**
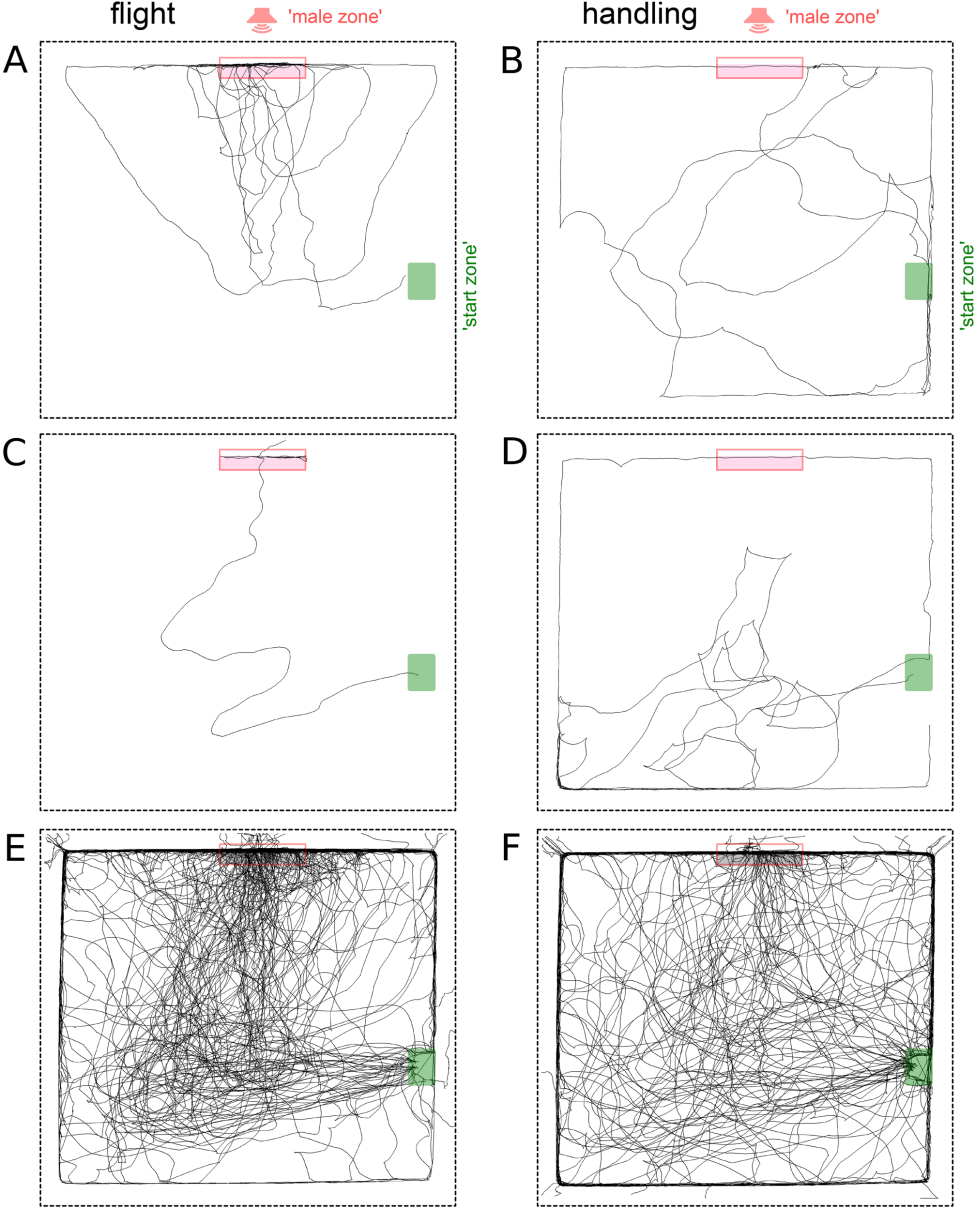
Visualized tracks of female crickets in the arena. **(A–D)**, individual tracks characteristic for 'flight' **(A,C)** and 'handled' (control) crickets **(B,D)**. **(E,F)** show the overlay of all individual tracks for 'flight' and 'handled' groups, respectively. The red box indicates the 'Male zone' inside the arena and including the lower part of the wall close to the loudspeaker (situated outside the arena and hidden behind the fabric wall). The green box shows the position of the home container, in which a cricket was put into the arena and which it was allowed to leave freely. The 'flight' crickets took a more direct path to the source of the calling song. See also Supplementary Figure S1 illustrating the distribution of cricket tracks in relation to the directional diagram of the speaker.

In the experiment with forced flight, 38 of 39 and 35 of 38 females in the flight and control (handled) groups left their home containers within the time of observation. These animals were used for further analysis. 35 of 38 ′flight′ female crickets reached the ′Male zone' in contrast to only 20 of 35 control females (p = 0.002; *X*^*2*^ test, Fig. 1). The Fig. 1 illustrates examples of individual tracks characteristic for the flight (a, c) and the control group (b, d) as well as the tracks from all animals pooled together in the flight (e) and the control group (f).

**Figure 2:**
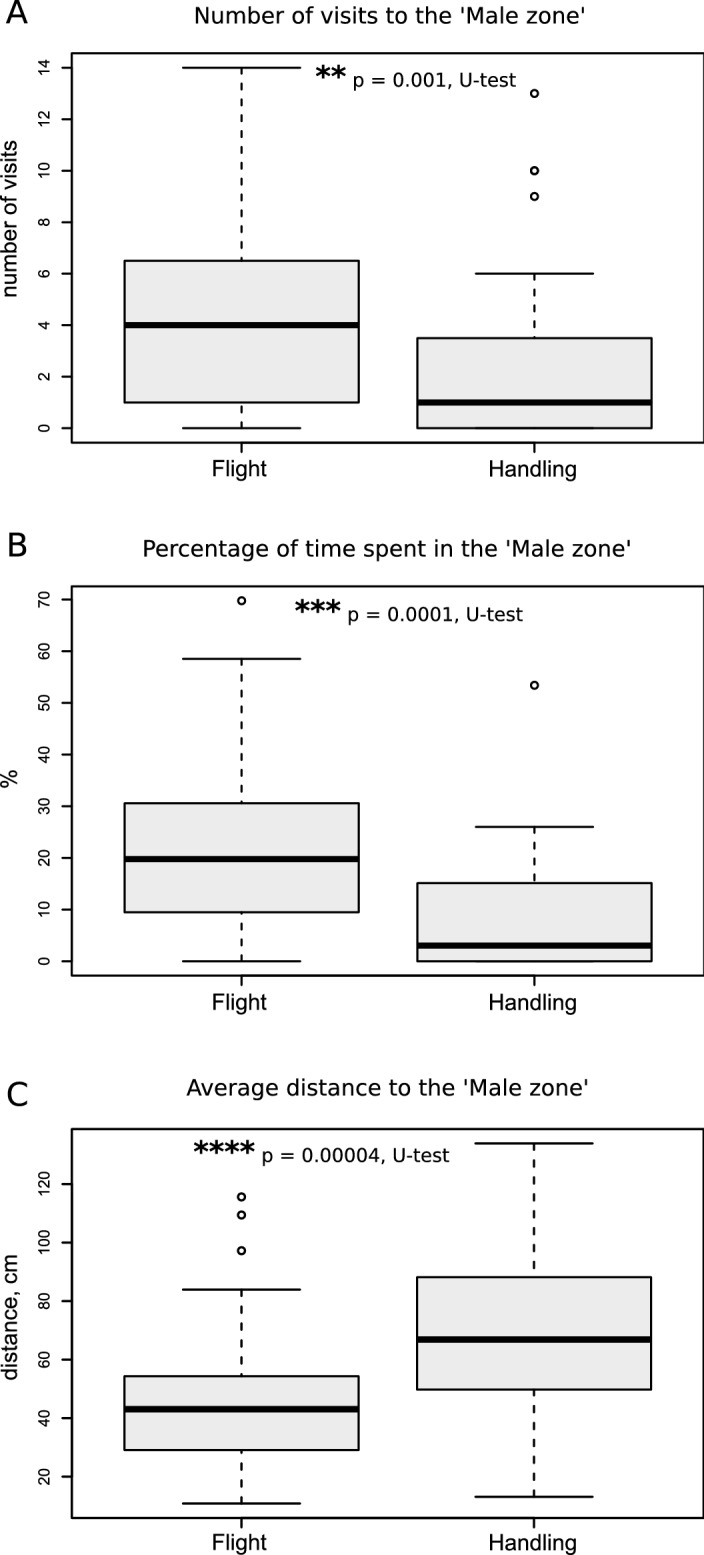
Quantitative parameters of sexual searching behavior in the 'flight' and 'handled' groups of female crickets. 'Flight' crickets visited the 'Male zone' more often **(A)**, spent relatively more time there **(B)** and had lower average distance, i.e. higher affinity, to the 'Male zone' **(C)**. The bold center time gives the median, boxes the interquartile range and whiskers 1.5 times the interquartile range. Data beyond this range ('outliers') are shown individually as points. Asterisks indicate the statistically significant differences according to the Mann–Whitney U test. Basic statistical analyses and drawings were conducted using the R^34^.

**Figure 3:**
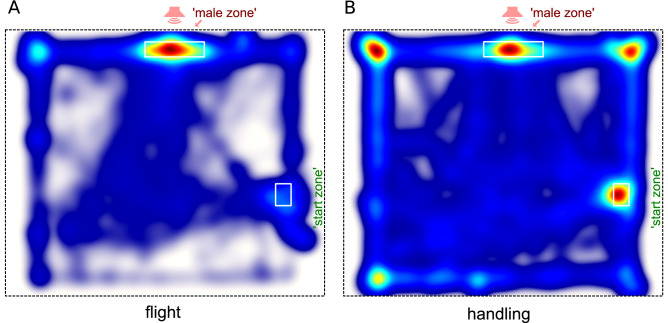
Heatmaps show the data from all crickets in the 'flight' group **(A)** and the handled group **(B)** normalized proportionally to the time spent by crickets in each site of the arena, from blue to red. 'Flight' crickets demonstrate clear preference for the ‘Male zone’ in contrast to the control group, showing preferences for the home container area and the corners of the arena as well. White boxes indicate the 'Male zone' close to the loudspeaker (that was situated outside the arena and hidden behind the fabric wall) and the position of the home container, in which a cricket was put into the arena and which it was allowed to leave freely.

The animals usually made several attempts to find the source of the calling song, thus revisiting the ′Male zone′, and 'flight' females did this significantly more frequently (p = 0.001; Z = 3.17; Mann–Whitney U test, Fig. 2a). They also spent more time in the ′Male zone′ (p = 0.0001; Z = 3.88; Mann–Whitney U test, Figs. 2b, 3). The average distance to the 'Male zone' was significantly lower (Z = 3.98, p = 0.00004, Fig. 2c), thus indicating the higher affinity of ‘flight’ crickets to this zone. ′Flight′ crickets figured out remarkably more often that they had to climb the wall to reach the loudspeaker (20 versus 7, p = 0.003). On the other hand, 'handled' crickets escaped the arena by climbing the wall in places other than the 'Male zone' during the first 10 min of the experiment more often than 'flight' females (n = 15 in contrast to n = 3, p = 0.002, *X*^*2*^ test). This behaviour of handled females occurred nearly as frequently as in the control unhandled group, in which 6 of 13 females escaped the arena before the end of the experiment. We suppose therefore, that escape was not a result of stress caused by handling.

**Figure 4:**
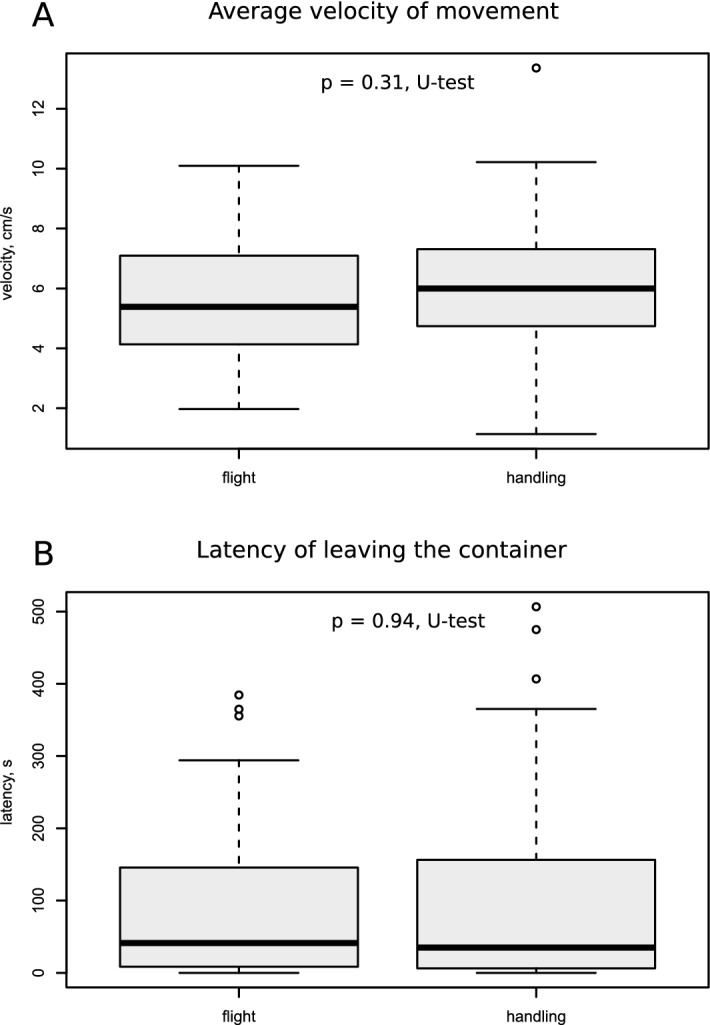
Quantitative parameters of motor behavior in the 'flight' and 'handled' groups of female crickets. The average velocity of locomotion in the arena **(A)** and the time they spent in the home container before leaving **(B)** did not differ. All symbols and notations as in the Fig. 2. Basic statistical analyses and drawings were conducted using the R^34^.

At the same time no significant differences were found either in the velocity of movement (p = 0.3; Z = − 1.21; Mann–Whitney U test, Fig. 4a) or in the latency time to leaving the home container (z = 0.67, p = 0.49, Mann–Whitney U test, Fig. 4b) between the flight and control groups of crickets.

**Figure 5:**
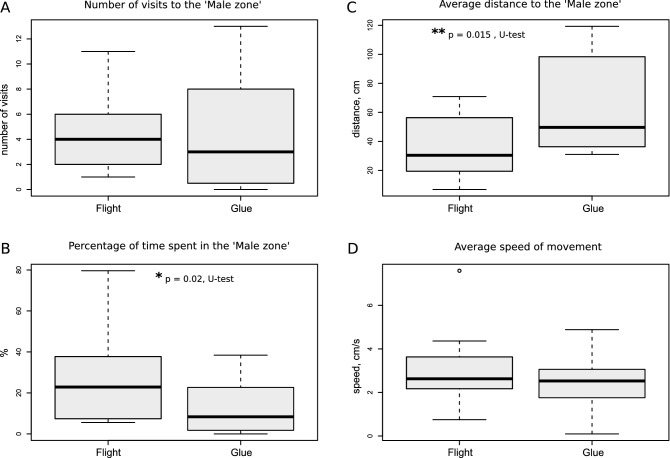
Quantitative parameters of behavior in the 'flight' and 'glue' groups of female crickets. The number of visits to the ‘Male zone’ **(A)**, the time spent in the ‘Male zone’ **(B)**, the average distance to the ‘Male zone’ **(C)**, the average speed of locomotion in the arena **(D)**. All symbols and notations as in the Fig. 2. Basic statistical analyses and drawings were conducted using the R^34^.

Similar results were obtained in the experiment with 'flight' crickets (n = 14) and control crickets that received glue at the thorax (n = 12). Although there was no significant difference in the number of visits to the 'Male zone' (Fig. 5a), all 'flight' crickets reached the 'Male zone' in contrast to 75% in the control, spent significantly more time in it compared to the control ones (p = 0.02; Z = 2.1; Mann–Whitney U test, Fig. 5a,b) and had lower average distance, i.e. higher affinity, to the 'Male zone' (p = 0.015, Z = 2.4; Mann–Whitney U test, Fig. 5c). Five 'flight' females in contrast to only one in the control group guessed to climb the wall to reach the loudspeaker. As in the previous experiment, there was no difference in the speed of locomotion between these groups (Fig. 5d).

**Figure 6:**
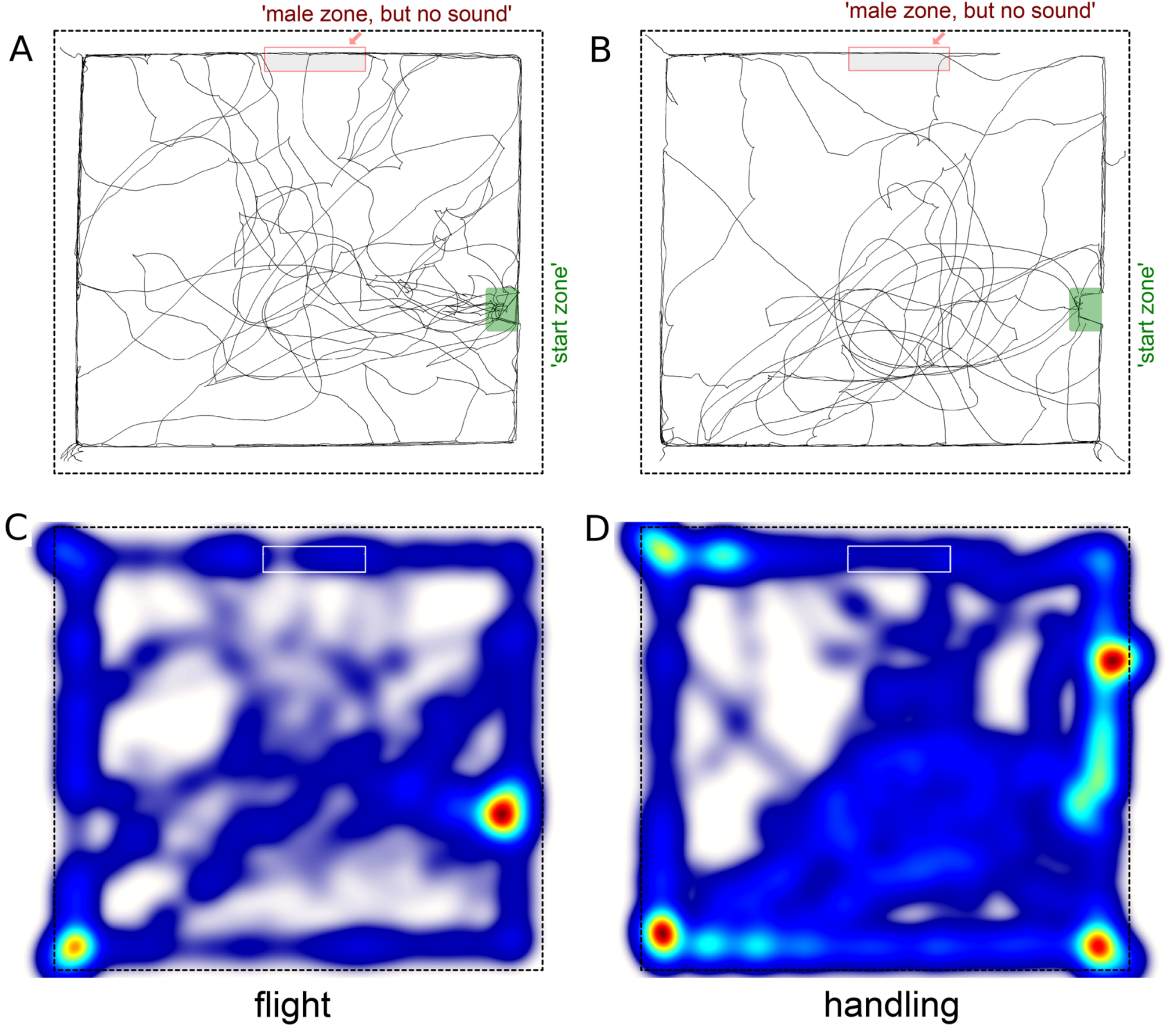
Visualized tracks of female crickets in the arena with the loudspeaker turned off. A and B show the overlay of all individual tracks for 'flight' **(A)** and 'handled' groups **(B)**, respectively. Heatmaps **(C,D)** show the data normalized proportionally to the time spent by crickets in each site of the arena, from blue to red. All symbols and notations as in the Figs. 1 and 3.

When there was no calling song, female crickets did not show a preference for the ′Male zone′, as can be seen in Fig. 6, depicting the tracks of female crickets (Fig. 6a,b) and the heatmaps normalized proportionally to the time spent by crickets in each site of the arena, in color scale from blue to red (Fig. 6c,d). There were no differences between the control and flight groups in the investigated behavioral parameters in these conditions. The mean speed of locomotion was 4.2 ± 2.4 in the flight group and 4.5 ± 3.2 cm/min in the control group (z = 0.04, p = 0.9), the mean number of visits to the silent ′Male zone′ was less than 1 in both groups: 0.55 ± 1 and 0.3 ± 0.7 (z = 0.39, p = 0.61). There were no occurrences of climbing the wall in the silent 'Male zone' neither in the control, nor in the experimental group.

## Discussion

Intense locomotion, such as running, flying or fast swimming, can bring an animal into a less familiar environment. Unpredictable conditions decrease the chances of survival and demand higher cognitive and physical abilities in order to navigate, avoid possible risks or reach a goal. It has been suggested that feedforward activation of physical and cognitive abilities following intense locomotion can be beneficial and even critical for survival in the novel environment. In other words, intense locomotion can be used as a predictor of novelty and activate mechanisms of biological and cognitive “preadaptation” to less familiar environments^20,21^. Indeed, stimulating effects of running on the brain function in humans and rodents have been repeatedly demonstrated^22–24^. Decreased anxiety^25,26^, augmented neurogenesis^22,27,28^, improved memory and learning^22,27^, facilitation of decision-making^20,21,29^ reported in numerous studies would be important or even crucial for surviving in the novel conditions. Recent studies reported behavioral benefits of intense locomotion for subsequent orientation in a novel environment in an evolutionarily distant animal, the mollusc *Lymnaea stagnalis*^20,21^, suggesting that this phenomenon might have deep roots in evolution.

In the life history of many insects, periods of intense locomotion such as flying precede mating behavior under natural conditions^1,8,15^. Short- and long-distance flights prior finding a partner may serve to decrease the inbreeding rate and to inhabit the novel areas. At the same time, getting into less familiar conditions might increase uncertainty that may, in its turn, severely decrease the competitiveness of intruders and, eventually, abolish all expected benefits of flight. It seems likely that biological mechanisms which may help to deal with the anticipated novelty and uncertainty could have evolved to protect the intruders and to stimulate migratory behaviors. Indeed, some evidence to this idea has already been found. In crickets, forced flying had been found to increase the aggressiveness in males, to restore the ability to fight and win in losers, and even to produce winner^2,3^. Flight activated courtship singing in male crickets and increased their mating success^4^. All these effects can be considered as promoting the competitiveness of animals after even a short period of flight.

Our findings clearly show that intense locomotion by itself affects subsequent behavior in a novel environment. Female crickets demonstrate a better ability to find a calling male by following the sound in a novel territory after a short period of forced flight. The improved ability to correctly locate and physically reach a male is evidenced by the higher number of animals that reached the ′Male zone′ in the arena and by the increased number of climbs up the wall to find the hidden source of the male song. The increased time spent in the ′Male zone′, the increased affinity to this zone measured as lower average distance from it and higher number of returns to this zone suggests higher sexual motivation and interest to the male song in females after the flight. Of course, an increased motivation and better results in reaching the hidden source of the calling song are likely to be connected.

The involvement of unspecific general activation in the above effects of flight seems unlikely: there was no difference in the latency to leave the container and in the mean speed of locomotion between the 'handled' and 'flight' females. We can also exclude a possible nonspecific increase in climbing activity after the flight because control females also demonstrated climbing, but in other parts of the arena. Finally, we did not detect the differences in the behavior between the control and 'flight' females in the same arena when the speaker was turned off. This finding points to sexual motivation as the main parameter affected by previous flying.

Previously, in males *G. bimaculatus*, a decreased responsiveness to startling stimuli had been documented after forced flight^3^. Although we found no direct evidence for the difference in the anxiety between the control and the 'flight' animals in the present behavioral tests, a decreased anxiety in the novel environment could potentially contribute to the effects observed in this study. The animals could freely choose when to leave their home containers placed in the novel environment, which, as we believe, reduced the number of stressing factors. Control crickets, although they left the container at the same latency as the 'flight' ones and went to explore the open arena, tended to escape from it, paying less attention to the calling song. Notably, this arena-escaping behavior was frequently observed not only in handled control crickets but also in the unhandled group. Therefore, it is unlikely that escaping behavior could result from stressing procedures such as handling or gluing.

Presumably, the intraspecific calling song could also indicate an area of 'cricket wellbeing' and thus point to the safer side of the novel territory. This advantage was still ignored by significantly higher number of the control females escaping from the arena. Therefore, the behavior of 'flight' females in novel circumstances can in general be considered more adaptive. Whenever the effect of flight on male-oriented goal-directed behavior partially depends on a decreased anxiety, flight clearly facilitates the release of sexual motivation and related cognitive and physical efforts.

During flight, crickets demonstrate positive phonotaxis to the intraspecific calling song^30^. In females, at least in the tethered flight experiments, a significantly sharper auditory tuning to a calling song was observed during flight in comparison to walking locomotion^31^. Similarly, the modulatory effect of flight on spontaneous electrical activity and evoked responses to visual signals of motion-sensitive sensory neurons has been reported in the blowfly *Lucilia *spp^32^. The application of octopamine agonist chlordimeform which mimics an increased octopamine release during flight produced similar changes^32^. It is unclear whether the above effect on the auditory system is also mediated by the octopamine modulation. It is unknown whether it lasts after the end of flying as do some other effects of intense locomotion. Potentially flight-induced auditory tuning could play a role in the improved ability to locate the source of the male song in our experiments.

Although there might be a difference in the behavioral effects of natural and forced flight, the obtained data in male and female crickets suggest that the benefits of sexual behavior activation after the flight might outweigh the costs, at least in this species. We consider the obtained effects as manifestations of proactive physiological adjustment to a novel environment that is likely to be reached as a result of flying. Activation of sexual behavior and related cognitive and physical abilities in a less familiar environment may help animals to cope with their decreased competitiveness and potentially decreased survival rate as intruders. These effects may also be a part of preadaptation to the unpredicted abrupt changes in the environmental conditions that would allow the cricket population to survive.

It remains to investigate whether flight exerts similar effects in familiar territory. Although we did not test the effect of flight on female behavior in familiar conditions, we suggest that the effects of intense locomotion are not likely to be strongly dependent upon the context. In conditions of novelty, cognitive and physiological activation after intense locomotion may indeed be crucial for survival, and that is why these effects may have appeared in evolution. However, in the cases when an animal was returned to or remained in familiar context after a bout of intense locomotion, these activating effects could be neutral to beneficial, and therefore are likely to persist independently of the context.

## Material and methods

### Experimental animals

Female, adult, sexually mature crickets *G. bimaculatus* DeGeer were taken after the final molt from the breeding colony maintained at the Koltzov Institute of Developmental Biology (Moscow, Russia). They were then kept in a separate colony without males in 60 × 50 × 55 cm containers with water and food (apples, carrots, salad, dry gammarus and wheat bran) ad libitum. The time of separation from males could vary from 14 to 20 days in different experimental sessions. A single experimental session contained only females that were collected simultaneously from the colony and kept for the same amount of time separately from males. One day before the experiment, they were isolated from each other in round plastic containers (diameter 93 mm, height 100 mm, made of opaque white plastic with transparent perforated lid) with free access to food. That period of isolation was expected to eliminate the differences between the animals related to their social experience^2–4,17,19^.

### Behavioral experiment

We developed a new behavioral paradigm to test sexual motivation and ability to locate the source of male song in a brightly lit spacious arena (150 cm × 150 cm, Fig. 1), which was meant to be a novel environment for a cricket raised in a laboratory colony. The floor of the arena was made of plywood with polyurethane matte non-slip coating. The 20 cm tall walls were made of white fabric stretched between the holders at each corner.

After the induction of flight or control procedures, a cricket was returned into the same individual plastic container in which it had been kept previously, for acute stress relief for 2 min. Then a container with a cricket was carried to the experimental arena, opened and gently placed on the side near the wall of the arena, opening towards the center of the arena, so that the cricket could freely leave. The duration of the experiment was limited to 10 min, but the experiment stopped earlier if a cricket escaped the arena by climbing over the wall.

The loudspeaker was positioned behind one of the walls, to the right from the cricket′s starting position (Fig. 1, Supplementary Figure S1). The loudspeaker (Teac TE-T15) powered by an integrated amplifier (Dynavox DA-30) with a high-pass filter aired the calling song of a male cricket, which was previously recorded in the colony and looped to provide a continuous stimulation program for the time of the experiment. The amplitude of the sound inside the arena was 85 dB SPL at 20 cm from the speaker, approximately the same level as when the sound was recorded in the colony. The speaker was positioned behind one of the walls, to the right from the cricket′s starting position (Fig. 1) as close to the surface of the arena as its diameter would allow, centered at a height of about 2.5 cm. To reach it a cricket needed first to ascend the wall and then to descend to the speaker outside the arena. We used the expression 'climb the wall' to describe this behavior. In most cases, however, we did not wait till a cricket descends as this would often lead to escape of a cricket, and removed it from the wall of the arena.

The speaker was mounted on a tripod and not attached to the arena in order to avoid the vibrations from it propagating through the arena floor, as crickets are known to pay attention to substrate vibrations accompanying a male's calling song^33^. We could not adequately mimick vibrational component of the song and thus decided to exclude it altogether. The speaker was turned on just before placing the container with a cricket into the arena.

The arena was illuminated from above with four 60 W bulbs to provide 140 lx at the center. A video camera (TheImagingSource DMK 23GV024) was placed 1.5 m above the arena. The activity of each cricket was recorded at 25 fps using IC Capture 2.2 software. The recordings were subsequently video tracked using Ethovision XT 13 software (Noldus, the Netherlands).

In the preliminary series (n = 13), naïve (preisolated, not handled for at least 24 h) crickets were put into arena in their containers. Further, to avoid multiple comparison procedures, crickets were randomly divided into two groups, experimental flight group and control group, and tested in alternation.

To induce flight behavior, a cricket was handled and glued to a holder by the upper side of the thorax. Flight behavior was evoked by suspending the animal for 3 min in an air stream produced by a fan, as in previous studies^2–4^. Detachment of legs from the ground may activate CPG for flying behavior in insects, however an additional wind stimulation produces more rapid and more sustained flight activation^2–4^. The control crickets were either handled (kept between fingers of experimenter) for approximately the same time as in flight group (3 min) as in^2–4^ or received a portion of glue at the upper side of the thorax. We did not test the possible effect of an air stream without suspending crickets, as no significant effect of this treatment had been demonstrated earlier^2^.

To make sure that visits to the ′Male zone′ were mainly caused by the male's calling song and not by occasional factors, an additional control experiment was performed with flight/handled crickets, as in the major experiment, the only difference being that the loudspeaker emitting the male calling song was turned off.

### Analysis of behavioral responses

We designated the 'Male zone' as a rectangular area 34 × 10 cm adjacent to the wall near the loudspeaker. For each cricket, we measured the following parameters:

- latency to leaving the container;
- average velocity;
- number of animals that reached the 'Male zone';
- number of visits to the 'Male zone';
- percentage of time spent within the ′Male zone' to the total time in the arena after leaving the container;
- average distance to the 'Male Zone'—the momentarily distance from the animal to the nearest border of the 'Male zone', averaged for the whole track; to calculate it we used a parameter 'Distance to zone' in the EthoVision XT software.
- the number of climbs over the wall, separately within the ′Male zone′ and outside of it.

A seemingly appropriate parameter, the time spent to reach the 'Male zone', was hard to estimate correctly due to the high number of crickets that escaped the arena before reaching the 'Male zone'.

### Statistical analysis of data

Basic statistical analyses were conducted using the R^34^ and STATISTICA software. The significance of differences was tested using the Mann–Whitney U test for variables such as the latency to leave the home cage, the mean speed of locomotion, the number of visits to the ′Male zone′, the time spent in the ′Male zone′. All values are given as the median and quartiles. The *X*^2^ test was used for comparing the number of animals that reached the 'Male zone' as well as for the number of climbs (the analysis was performed using the online application at http://medstatistic.ru) (Suppl Information).

### Ethics

All experiments complied with the Russian and international laws.

## Supplementary Information

Supplementary Figure S1.

## Data Availability

The data are available in 10.6084/m9.figshare.12453485.
