# Supplementary material for: Previous flight facilitates partner finding in female crickets
Source: Sci Rep. 2020 Dec 18;10:22328. doi: 10.1038/s41598-020-78969-w (PMC7749130; doi:10.1038/s41598-020-78969-w)
Supplement: Supplementary file 1 — Supplementary Figure S1. [file 41598_2020_78969_MOESM1_ESM.pdf]

SUPPLEMENTARY INFORMATION FOR

Previous flight facilitates partner finding in female crickets

AUTHORS

Maxim Mezheritskiy[1, †], Dmitry Vorontsov[1, †], Dmitry Lapshin[2], Varvara Dyakonova[1,*]

1, Koltzov Institute of Developmental Biology of the Russian Academy of Sciences, Moscow; 2, Institute for Information Transmission Problems of the Russian Academy of Sciences, Moscow

† These authors have contributed equally to this work

** Corresponding author:*

Varvara Dyakonova

Koltzov Institute of Developmental Biology of RAS, Vavilov Str. 26, 119334 Moscow, Russia. Phone 7-499-1355254, Fax 7-499-1358012, e-mail : <dyakonova.varvara@gmail.com>

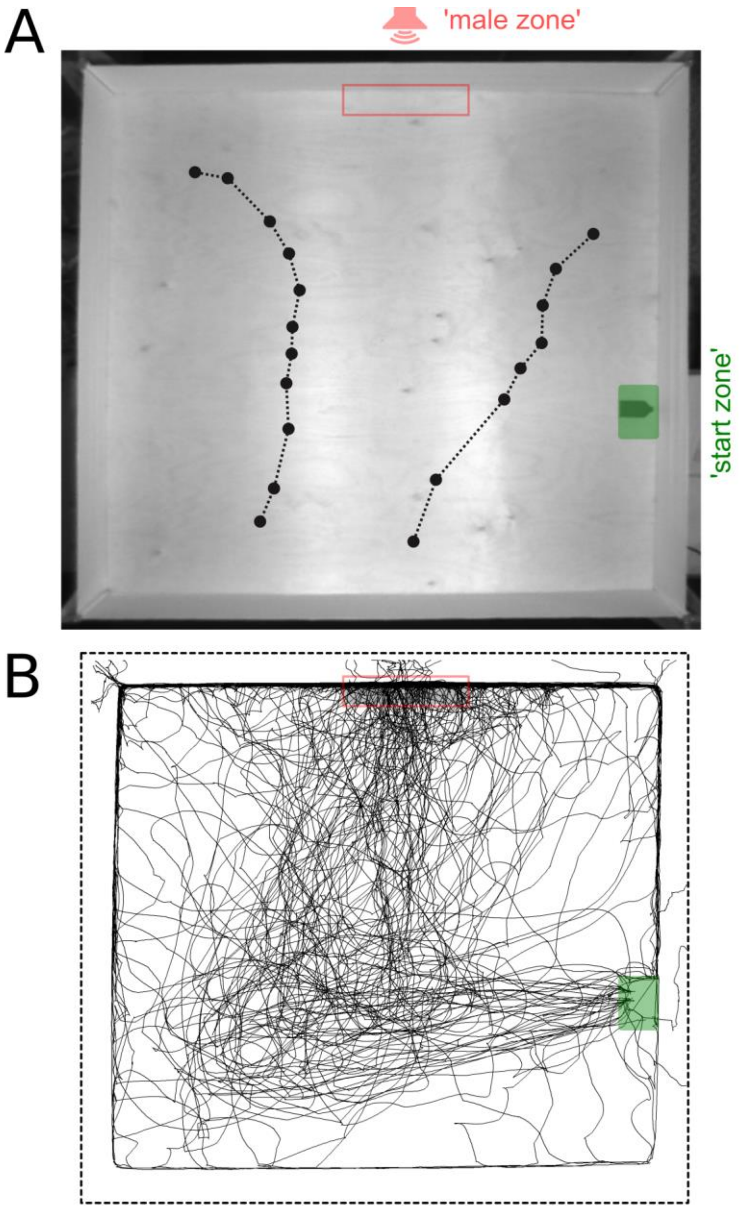

Supplementary Figure S1: Distribution of cricket tracks in relation to the directional diagram of the speaker. A. Directional diagram of the speaker in the experimental arena. Black filled circles show the points of equal level of sound (5 kHz, 60 dB) measured 3 cm above the arena floor by CEM DT-805 sound level meter. B. Overlapped tracks of crickets from the 'flight' group. Image shows the tendency towards two different strategies in reaching the 'Male zone' (indicated by red box): some crickets turned to the source of sound after reaching the maximum amplitude of sound, some kept moving until the level of sound decreased, and then turned to the source. The position where the home container was placed ('start zone') indicated in green.
